# Family Caregivers’ Experiences and Coping Strategies in Managing Stroke Patients during the COVID-19 Pandemic: A Qualitative Exploration Study

**DOI:** 10.3390/ijerph19020942

**Published:** 2022-01-14

**Authors:** Muhammad Iqbal Haji Mukhti, Mohd Ismail Ibrahim, Tengku Alina Tengku Ismail, Iliatha Papachristou Nadal, Sureshkumar Kamalakannan, Sanjay Kinra, Kamarul Imran Musa

**Affiliations:** 1 Department of Community Medicine, Health Campus, School of Medical Sciences, Universiti Sains Malaysia, Kubang Kerian 16150, Malaysia; muhammadiqbal@student.usm.my (M.I.H.M.); dralina@usm.my (T.A.T.I.); drkamarul@usm.my (K.I.M.); 2 Department of Non-Communicable Disease Epidemiology, Faculty of Epidemiology and Population Health, London School of Hygiene & Tropical Medicine, Keppel Street, London WC1E 7HT, UK; iliatha.papachristounadal@lshtm.ac.uk (I.P.N.); Suresh.Kumar@lshtm.ac.uk (S.K.); sanjay.kinra@lshtm.ac.uk (S.K.); 3 Department of Social Work, Education and Wellbeing, Faculty of Health and Life Sciences, Northumbria University, New Castle NE7 7XA, UK

**Keywords:** COVID-19 pandemic, stroke, family caregivers, burdens, experiences, coping strategies, public rehabilitation centers

## Abstract

Background: Stroke is a chronic disease that requires stroke survivors to be supported long-term by their families. This is especially because of the inaccessibility to post-stroke rehabilitation outside hospitals. The Corona Virus Disease 2019 (COVID-19) crisis and the pandemic restrictions in Malaysia are expected to exponentially increase the demand from family caregivers in supporting stroke survivors. Thus, this study aims to explore the burden, experience, and coping mechanism of the family caregivers supporting stroke survivors during the COVID-19 pandemic. Methodology: A phenomenological qualitative study was conducted from November 2020 to June 2021 in Malaysia. A total of 13 respondents were recruited from two public rehabilitation centers in Kota Bharu, Kelantan. In-depth interviews were conducted with the participants. Comprehensive representation of perspectives from the respondents was achieved through purposive sampling. The interviews were conducted in the Kelantanese dialect, recorded, transcribed, and analyzed using thematic analysis. Results: Three themes on burdens and experiences were identified. They were worsening pre-existing issues, emerging new issues, and fewer burdens and challenges. Two themes on coping strategies were also identified. They were problem-focused engagement and emotion-focused engagement. Conclusions: The COVID-19 pandemic has changed the entire system of stroke management. While family caregivers mostly faced the extra burden through different experiences, they also encountered some positive impacts from the pandemic. The integrated healthcare system, especially in the era of digitalization, is an important element to establish the collaborative commitment of multiple stakeholders to compensate burden and sustain the healthcare of stroke survivors during the pandemic.

## 1. Introduction

Non-communicable illnesses accounted for seven of the ten major causes of death globally in 2019, with stroke coming in second [1]. The same trend was observed in upper and upper-middle-income countries [1]. In 2016, the Global Burden of Diseases, Injuries, and Risk Factors Study (GBD) highlighted stroke as the second-leading cause of death, with approximately 5.5 million deaths, 116.4 million disability-adjusted life years (DALYs), 13.7 million new stroke cases, and a prevalence of 80.1 million cases. The trend from 1990 to 2016, East Asia showed an increment of age-standardized stroke incidence by 4.9%, thus manifesting the burden of stroke as relatively significant [2]. Globally, by 2040, stroke is expected to be the second-most common disease after ischemic heart disease [3].

According to the Annual Report of the Malaysian Stroke Registry 2009–2016, the stroke mortality rate had increased in Malaysia from 2010 to 2016 [4]. In 2017, stroke was the third leading cause of premature death. Consistently, in 2019, stroke was the most common cause of death and the second-most combining death (years of life lost) and disability [lived with disabilities as measured by Disability-Adjusted Life Years (DALYs)] [5]. According to the National Health Morbidity Survey 2011 (Volume III), the prevalence of stroke in Malaysia was 0.7 percent (20,966 cases), while the state of Kelantan was reported with 1338 stroke cases and at a prevalence rate of 0.5% [6]. A recent local study has shown 14,396 stroke cases reported across 11 states in Malaysia from 2012 till March 2019. Kelantan was ranked as the fourth state with the highest cases in Malaysia (1620 cases) after Terengganu (6744 cases), Sarawak (2340 cases), and Pulau Pinang (1754 cases) [7].

The global pandemic of COVID-19, which World Health Organization (WHO) officially announced on 11 March 2020 [8], has significantly impacted stroke caregiving, especially movement control order (MCO). Malaysia Government Movement Control Order was first imposed on 18 March 2020 to effectively manage disease transmission under the Control and Prevention of Infectious Diseases Act 1988, and the Police Act 1967 [9,10] has limited the rehabilitation and treatment process. Retrospectively, an abundance of studies was established to observe stroke burden upon survivors [11,12,13], caregivers [14,15], and the healthcare system [16,17,18]. Regardless, only a few studies have been linked to such a global health crisis. Despite the fact that several studies have shown a significant decline in stroke admissions during the COVID-19 pandemic [19,20,21,22,23], the findings have not revealed the cause, and there is currently a scarcity of data to explain the decline. Moreover, only a few published resources addressed the stroke burden of family caregivers during the COVID-19 pandemic [24,25], but none linked to the local context of Malaysia. The well-being of caregivers will determine the survivability of the stroke patient.

Therefore, the aim of this study is to explore the impact of the COVID-19 pandemic on stroke family caregivers by exploring their experiences, burdens, and coping strategies.

## 2. Materials and Methods

### 2.1. Study Design

This study applied a qualitative study design with an explorative phenomenological approach to investigate the individual’s perspective and how they perceived or interpreted the stroke caregiving during the COVID-19 pandemic [26].

### 2.2. Study Participants

This study was conducted from November 2020 to June 2021 at two main public rehabilitation centers: Pusat Pemulihan & Rehabilitasi Hospital Raja Perempuan Zainab II (HRPZ II) and Hospital Universiti Sains Malaysia (Hospital USM) in Kota Bharu, Kelantan. The stroke family caregivers act as the reference population. The source population was the family caregivers managing stroke patients who registered at the Hospital Universiti Sains Malaysia (Hospital USM) or Hospital Raja Perempuan Zainab II (HRPZ II), Kota Bharu, Kelantan, and fulfilled the following criteria: the caregiver must be a spouse or other family member or a relative of the stroke patient who is 18 years old or older and willing to share their experiences. The stroke patients were selected based on Malaysia Clinical Practice Guideline, defined as the clinical state that has no apparent aetiology other than vascular origin and is primarily associated with atherothromboembolism, intracranial small vessel disease, cardiogenic embolism, and other vascular conditions [27]. Otherwise, stroke presentation caused by transient ischemic attacks, tumors or malignancies, spinal cord injuries, and motor vehicle accidents would be excluded.

In general, no scientific method exists for determining the sample size of qualitative research [28], but the most important factor in determining the sufficiency of data in qualitative research is once saturated [29]. According to the literature, a group of 12 to 26 people appears to be adequate in terms of practicality [30]. In parallel, a total of 13 respondents participated in this study until data saturation was reached, which is a necessary component of data sufficiency [31].

### 2.3. Data Collection

An interview guide was developed and verified by the expert team members before the process of selecting participants began. The maximum variation or heterogeneous sampling is a purposive sampling technique [32] that was applied purposely to assure a variety of variables and to sample the registered patients diagnosed with stroke by neurosurgeons or neuromedicals and physiotherapists at any of the two named centers. The clinical teams first identified the appropriate participants while the patient and family caregivers were present at the appointment. The researcher was then contacted to obtain consent in the same setting to avoid the possibility of dropout if the meeting is rescheduled in another setting. The participants were briefed in detail and informed of their right to withdraw at any time during the study process. Written consent was obtained before the interviews, signed by participants. The in-depth interviews were conducted and recorded with permission by using a digital voice recorder. Two pilot interview sessions were conducted before the major study, which resulted in some revisions and improvements to the interview guide. Each interview lasted 30 to 45 min, and for each session that provided significant information, a revision was performed if necessary. Eventually, all the information was entered into the data analysis. Overall, the three main questions were asked “What is your typical role in managing stroke patients, especially during the COVID-19 pandemic?”, “Could you please explain more regarding your previous experiences during the caregiving of stroke patients and how much it was affected by Movement Control Order (MCO)?”, “How do you cope with all the difficulties you faced while managing stroke patients in the pandemic situation?” Appendix A (Table A1) has a detailed breakdown of the primary and probing questions. The interview sessions were established in the local Kelantanese Malay dialect. The data collection process was terminated once the data reached saturation, whereby no new themes, coding, or data were handed over to the next sequential samples, as stated by O’Reilly and Parker [33].

### 2.4. Data Analysis

Overall, 13 interviews were conducted with the last two additional sessions suggesting that no new data was generated, assuring that the data obtained were sufficient [34]. According to Braun and Clarke [35], this study used a six-phase thematic analysis: (i) familiarizing oneself with data, (ii) generating initial codes, (iii) searching for themes, (iv) reviewing themes, (v) defining and naming themes, and (vi) producing the report. The first author transcribed the recorded interviews verbatim, and the transcripts were rechecked by other authors. The entire 13 interviews were transcribed in Kelantanese Malay dialect. The first three interviews were read and re-read to become familiar and have a better understanding of the content, generating the initial codes. Then, the first author identified the data extracts in line and analyzed them independently to code the data extracts from all interviews. This was performed by writing notes and codes in the margins of the extracts, and subsequently, a meeting involving all the authors was organized to discuss the findings from the extracted data until there was a consensus. These similar processes were repeated for the other interviews. The subsequent meetings were held with all the authors several times to revise, discuss, and agree on the coding. Themes were searched by first organizing the codes into the potential subthemes. A thematic map was created to form themes based on the extracted codes and subthemes. The sense of significance and relationship among different themes were defined and named to represent the meaning, discussed, and reviewed among authors until agreement was reached. The final report of data extraction related to the research question and literature was the best final product of analysis.

### 2.5. Rigors and Trustworthiness

The four requirements to grow trustworthiness and establish rigors were credibility (internal validity), transferability (external validity/generalizability), dependability (reliability), and confirmability (objectivity) [36]. These attempts to prevent confirmation bias occasionally resulted in the “sense of adequacy” concerning data saturation. To establish credibility, the researchers performed “member checks” by having participants re-read transcripts, performed triangulation such as written medical records, information drawn from respective healthcare providers, self-observation, and regular discussions, conducted the pilot test of the interviews, and emphasized the researcher’s background, qualifications, and experience as the core competency while attending qualitative research expert talk. Regarding transferability, the fieldwork was presented in sufficient detail, assisting the understanding that guided the other readers [37]. The study processes were reported in detail to address the dependability issue, enabling readers to assess research procedures [38] and to frame a prototype for future researchers to repeat or gain identical findings. The operational details of the data collection process established involved recorded interviews, repetitive listening, coded data from a widen-to-narrow approach, cross-checked transcripts and codes, series of discussions till the final consensus, and reflective appraisal to assess the effectiveness of the process taken. In contrast, confirmability was attained by maintaining reflexivity, avoiding personal biases in data analysis, freeing participants to express thoughts or feelings without comments or gestures-made responses, and closely monitoring supervisory team members to keep the researcher focused on objectives.

### 2.6. Ethical Consideration

Ethical approval was granted by the Medical Research and Ethics Committee (MREC), Ministry of Health Malaysia [NMRR-20-351-53369 (IIR)], and the Human Research Ethics Committee, USM (JEPeM code: USM/JEPeM/20010031). The participation was voluntary, and the participants were informed beforehand that they had the right to stop the interview, withdraw, or refuse to answer without being penalized. The specific identification numbers stored on the recording labels were kept confidential and secured.

## 3. Results

### 3.1. Characteristics of Respondents

Table 1 shows the characteristics of family caregivers and the diagnoses of stroke survivors. The 13 people who took part in the survey came from a wide range of backgrounds.

Three themes emerged on burdens and experiences, whereas two themes emerged on the coping strategies. Several subthemes were derived, as exhibited in Table 2 and Table 3, respectively. The initial codes for each subtheme are detailed in Appendix B (Table A2 and Table A3).

### 3.2. Theme 1: Worsening Pre-Existing Issues

Since the pre-pandemic period, the caregivers were already struggling to provide care. The unsettling pre-existing issues and the presence of the COVID-19 pandemic were crucial factors that worsened the burden of caregivers. Whether the issues have been adequately addressed or, these burdens continue.

#### 3.2.1. Subtheme: Impact on Social Life

One respondent experienced an impact on her social life. She lacked time during the pre-pandemic, and her situation worsened with the implementation of Movement Control Order (MCO) during the pandemic. Her situation affected the management of the patient

*…MCO causes more problems to me… before this, should I have time, I could be with my friend, I could go to my friend’s house, sharing my problem… But now, due to MCO, it all seems to be so difficult and restricted…*

[R1, 24, daughter]

The COVID-19 pandemic prohibits any mass gathering, and to some extent, it limits the family caregivers from getting help from the neighborhood when necessary in delivering caregiving tasks.

*…MCO makes things so difficult… we could not ask for help from our neighborhood because there was a restricted movement order…even my aunt also could not come to visit my father…I felt so distressed; after all, I could not manage my parents for bathing or toileting.… Before the COVID pandemic, I had no problem because many people came to help me. My aunt always helped me to change the diapers and cook… But now, nobody comes…*

[R6, 25, son]

#### 3.2.2. Subtheme: Formal Obligation Responsibilities

The Movement Control Order (MCO) implementation during the COVID-19 pandemic led the family caregivers to struggle with the additional work demands, especially those employed as frontline workers. This further increased the burden of the family caregivers besides the existing challenge of the caregiving task on the stroke patient. A respondent said:

*…It’s tough during the COVID pandemic because I need to work outside the office; I’ve to take care of the roadblock… I’m tired because, by noon, I have to return home to shower both of my stroke parents… I don’t have enough time to rest. I am scared if I fall asleep, I will not be able to give enough care to them.*

[R6, 25, son]

Another respondent said:

*…My mom, for example, bounded with her duty at the office.…before pandemic she could easily drive us (me and my father) to the hospital, but during the pandemic… her working schedule seemed unpredictable… initially, she said she can go, but later on she said she can’t because she needs to urgently attend the court hearing…my father’s appointment frequently needs to be rescheduled…*

[R1, 24, daughter]

#### 3.2.3. Subtheme: Tight Appointment Schedules

Until the interview sessions were held, MCO 2.0 was introduced by the Malaysian government just after the recovery phase of MCO 1.0, implying the different changes of the previous setting. This was a factor that influenced the sentiment of a respondent to compare the discrepancy in the appointment schedule that was arranged between the two phases of MCO. The implementation of MCO 1.0 was considered firmer and rigorous than that of MCO 2.0, whereby most sectors were affected by the obligations made. Healthcare was one of the sectors affected unavoidable, whereby a respondent claimed that during MCO 1.0, the frequency of the appointment schedule was fewer than before MCO. However, during MCO 2.0, a multidisciplinary team arranged the appointment schedule more tightly to compensate for the postponement of appointments during MCO 1.0. One respondent expressed:

*…Previously (MCO 1.0), the appointment was held twice a month, and now (MCO 2.0), it occurs twice a week.…to me, it is difficult to handle if the appointment is made too frequent… because I stay in an army camp…work as an administrator, makes it difficult to leave the job frequently during the pandemic because the authority limits the number of workers per day… I am in a dilemma, to fulfill the need of my parent and at the same time to make sure my job does not interfere with the frequent appointments…*

[R6, 25, son]

### 3.3. Theme 2: Emergence of New Issues

With the presence of the COVID-19 pandemic, undeniably, some additional challenges arose along the route. Because the difficulties were not visible during the pre-pandemic period, the caretakers were unfamiliar with the challenges. However, with the multiple current demands due to the current condition, family caregivers need to try their best in caregiving stroke patients. Therefore, the “emerging of new issues” theme differs from the previous “worsening pre-existing issues” theme. This theme is also the inability to perform the activities that the respondents supposedly managed to do during the pre-pandemic.

#### 3.3.1. Subtheme: Restrictions in Mobility Indoors and Outdoors

Most of the respondents experienced limited mobility during MCO compared to the pre-pandemic period. This is shown in the responses below:

*…COVID pandemic does give a significant difference… before the COVID strike, we used to go to our son’s house… and sometimes, I would bring him for vacation with other family members…but now we need to stay safe at home…*

[R10, 54, wife]

However, as for another respondent, mobility limitation would consequently contribute to the increment of burden and less enjoyment with pleasurable activities.

*…To say what is the difference, basically, the pandemic gives additional burden to me… I have no time to get rest… I can’t go for a walk… I could not enjoy my life… spending more time at home means I have to deal with my wife more frequently…*

[R11, 66, husband]

Nevertheless, limited mobility would delay the recovery process of the stroke patient as well:

*…Due to COVID, our traveling to seek alternative treatment is interrupted… I could not bring my husband for massage, as before this we used to bring him for traditional massage daily…*

[R12, 57, wife]

#### 3.3.2. Subtheme: Changes in Health Care Services

A few issues were identified under this subtheme. There were rearrangements or postponement of appointments, limited participation of family caregivers in the patient’s rehabilitation process, and long waiting time. This is shown in the response below:

*…There are no huge differences except that the postponement of the appointment dates occur more frequently… it creates confusion sometimes.*

[R6, 25, son]

Besides the issue related to the rearrangement of the scheduled appointment, the COVID-19 pandemic would lead to a new issue regarding the changes in healthcare services, such as limited participation of family caregivers in the patient’s rehabilitation process in the hospital. A respondent said:

*…I think it is more comfortable before the COVID-19 pandemic… during pre-pandemic, I would be able to sit next to him assisting the physiotherapist… but now, I can’t… the hospital has limited the number of visitors to reduce the crowds, so I can’t participate… I can’t see how he goes about to perform his practices…*

[R3, 44, wife]

#### 3.3.3. Subtheme: Limitation due to Strict Standard Operating Procedures (SOP)

Before the pandemic era, healthcare services were operated with no limited numbers of patients with visitors. There was no issue on physical distancing, the requirement to wear a mask, or mandatory temperature checks. During the pandemic, the Malaysian government has endorsed a strict SOP to curb the transmission of disease that includes wearing masks, physical distancing, reducing numbers of patients and visitors per session in confined space and a need to register at any entering point using QR code [10]. The unfamiliarity with the process caused the time-consuming process of obtaining healthcare delivery services. A respondent said:

*…Before we were allowed to enter the clinic, we need to check our body temperature, scan the QR code, sometimes if we forgot to bring our phone, we need to write our name, etc…. all these cause significant delay especially when you are with a stroke patient…*

[R1, 24, daughter]

#### 3.3.4. Subtheme: Additional Patient-Care Responsibilities

The scheduled appointment was rearranged and postponed frequently due to Movement Control Order (MCO) as one of the control measures in healthcare services to prevent the transmission of COVID-19. Thus, the roles of care that physiotherapists partly bear, especially during in-patient care, were then undertaken by the family caregivers while at home. A respondent said:

*…The appointment during MCO is sometimes rescheduled and less in number, sometimes canceled, so we have more activities at home… so, I need to re-arrange the therapy’s schedule by myself… I need to do the therapy based on what I know, and sometimes I watch YouTube for help…”*

[R7, 35, wife]

The family caregivers were motivationally driven, as they were involved in the patient’s home exercises and did not let them perform by themselves. This extra role played by family caregivers would restore the home exercises efficiently, which physiotherapists would monitor once they attended appointments during pre-pandemic.

*…I need to focus on his hand… the hands need to be properly massaged so that the fingers do not go stiff… if I let him perform by himself, sometimes he would do it wrong…*

[R3, 44, wife]

#### 3.3.5. Subtheme: Constraints of Supportive Persons

Some of the family caregivers relied on other supportive persons in the caregiving of the stroke patient. Thus, the supportive persons belong to social support. Their limitations, for instance, the incompetency to drive a vehicle or even with the capability but lacking a driving license, made the patient and the primary caregiver depend on the availability of the respective person. It can be understood, especially when they were associated with their formal works. During the COVID-19 pandemic, the constraints that emerged from specific demands of their workplace, modification of work procedures, changes of work schedule, extra duty, and limitations on human resources significantly impacted the caregiving of stroke patients. A respondent expressed:

*…Because I could not drive, so someone else needs to drive us to have therapy, usually, my mum will do it and she needs to take time off from work on that day…. during COVID, mum was allowed to do work at home and her working schedule was more flexible…but… if her colleague is on leave, mom needs to replace her… it’s tough… So, she needs to be informed earlier the therapy schedule… because she needs to plan her working schedule properly…*

[R1, 24, daughter]

#### 3.3.6. Subtheme: Financial Constraints

As many other impacts were perceived during the COVID-19 pandemic, financial constraints were not an exception, and even the financial issue was highlighted as a major and significant burden to most of the family caregivers of stroke patients since pre-pandemic circumstances. However, this study aimed to focus solely on the impact of the pandemic, thus referring to the differences between pre-and during the pandemic era regardless of the financial status of both family caregivers and stroke patients in the previous condition. A respondent stated that her financial status was markedly affected by the presence of the COVID-19 pandemic. Though it had no direct impact on her, the COVID-19 pandemic affected her family members as the source of her income. Thus, this pandemic’s chain effects overtake each individual or group of persons and, in turn, the family caregivers of stroke patients as consequences.

*…Indeed yes…our financial status is affected during the COVID pandemic… It is because… we’re relying upon financial aids from our children (start to cry)… they used to give us the support… but, now life is too hard…my second child needs to be close to her tuition center…therefore less funding was given to us…*

[R10, 54, wife]

The COVID-19 pandemic affected not only the family’s financial sources but also the cost of necessities increased during the COVID pandemic.

*…During this pandemic, we had extra expenses… before the COVID strike, things were cheaper and easy to get…but now, it’s becoming more expensive, it is very costly especially when we have a disabled family member to take care of…*

[R9, 18, daughter]

### 3.4. Theme 3: Fewer Burdens and Challenges

In contrast to the previous two themes, this theme was uniquely identified from the positive perspective of some of the respondents on the impact of the COVID-19 pandemic. This is because the presence of the pandemic brought some favorable conditions to the family caregivers and facilitated their burdens in some specific ways. In other words, the existence of the COVID-19 pandemic would lessen some burdens and challenges to family caregivers.

#### 3.4.1. Subtheme: Improvement in Hospital Logistic Issues

One of the issues that were constantly a dilemma to most respondents was the hospital logistics, especially the parking space. This was not a new issue for most of them and has remained a problem since the pre-pandemic era. The high burden of diseases every day contributed to many patients seeking treatment and consequently to their caregivers and visitors to the hospital, limiting the hospital car parks available. Throughout the pandemic, the hospital took some control measures to reduce the attendance of patients and caregivers to avoid crowds and strictly limit the number of visitors. This would preferably be attributed to the availability and accessibility of hospital car parks.

*…The only problem before the COVID pandemic is the availability of parking lots in the hospital… but during this COVID pandemic, we are blessed….this problem is no longer a big issue…*

[R3, 44, wife]

*…It’s quite easy to park the car during the COVID pandemic, fewer people visit the hospital means more parking lots are available.*

[R10, 54, wife]

#### 3.4.2. Subtheme: Strengthening the Family Bonding

The COVID-19 pandemic and Movement Control Order (MCO) allowed more time for the respondent to be at home together with the patients. Hence, family bonding was strengthened, and responsibilities were shared between family caregivers and support persons, which lessened the burden only on the supportive person even during the current disastrous condition. For example, one respondent claimed that:

*…COVID pandemic means our movement is restricted…I just spend time with her… that’s the best time to take care of her … at least, to lessen the burden of my in-laws… so, I can take turn to give the quality care for her…*

[R2, 43, husband]

*…COVID-19 means I can spend more time with my husband… I could assist him to do his therapy at home… able to provide the best care for him without thinking to go out and spend time with others…*

[R3, 44, wife]

There are two major themes derived from the analysis of coping strategies. This manifestation reflected the two different ways stroke family caregivers overcame difficulties during the pandemic of COVID-19: (i) problem-focused engagement and (ii) emotion-focused engagement. The coping strategies were committed to the important role of the supportive person, adaptations made to the changes of healthcare services, and facilitated mobility during the pandemic, which was derived under the theme of problem-focused engagement. In contrast, the subthemes of obeying strict Standard Operating Procedure (SOP) of new norms and passive response to the burdens and challenges were formed under the theme of emotion-focused engagement. The caregiver’s coping strategies were narrated in detail in the following sections.

### 3.5. Theme 1: Problem-Focused Engagement

#### 3.5.1. Subtheme: Adapting the Available Support

One of the challenges that were demonstrated earlier was the constraints of the support person. Undeniably, the primary family caregivers would encounter challenges in caregiving many aspects of their capacities. Thus, they need support from others to help them facilitate caregiving tasks on stroke patients. To some extent, the support person would face obstacles, particularly if they were working. The demands of work during the COVID-19 pandemic would unconditionally augment the burden of the support person. Therefore, to cope with this hardship, the respondents would seek help and commitment from another person who could support their caregiving tasks. This indicates that the importance of the role of the surrounding people contributes to indirectly facilitating the caregiving of stroke patients. One of the respondents said:

*…During the COVID pandemic, my brother often drives us to therapy as previously, my mom did it…it is just because his employer allows the employee to take maximumfour4 days of leave in a month, therefore with the current biweekly appointment of our father, we might not have any problem to send the father to the therapy…*

[R1, 24, daughter]

Most of the respondents would face limitations in their leisure activities due to the restriction during Movement Control Order (MCO). Thus, the caregivers and stroke patients tend to spend the whole time being only at home. This would trap them from going anywhere, and, as a consequence of the long-term effect, it would cause a stressful environment for both patient and family caregivers. In contrast, for a respondent, she would try to bargain with this limitation. Although she might constantly choose to go nowhere, like the other family caregivers, she actively accompanies the patient for leisure activities as a coping strategy during this pandemic. This coping strategy would be performed in her way but cautiously not to break the rule, and this would help her care for the stroke patient sustainably in the long run. She stated:

*…Sometimes I bring my husband for a walk in the early morning at the nearby garden when everybody is still sleeping, when the sun rises, we start to go home… even walking on the beach during late evening when there are fewer peoples around…this could release my stress and his stress …*

[R12, 57, wife]

#### 3.5.2. Subtheme: Adaptation to the Changes of Healthcare Services

Regarding adaptation to the changes in healthcare services during the COVID-19 pandemic and limitations in mobility, most of the respondents would call for an appointment. This would help them keep in touch with the therapy sessions even during the pandemic crisis. They stated:

*…Have you missed the appointment? (interviewer asked)…(the respondent answer) Nope… we never missed the appointment; during COVID, we would call the rehabilitation center first, asking the staff over there… if they said no need to come, then we will not come and a new date was given… so we did not waste our time and it was less stress…*

[R8, 59, father]

The medications for treatment were different from the physiotherapy sessions. Some respondents would take the initiative to call and continue the medication by simply updating the current condition of stroke patients. A respondent said:

*…If the medication is almost running out but the appointment date is not yet reached, I will call the nurse at the rehabilitation centre; she then would inform a doctor…The doctor prescribed the online prescription… so, no need to come, I’d pick up the medications by myself…*

[R2, 43, husband]

#### 3.5.3. Subtheme: Permission to Mobilize during the Pandemic

As stated before, the restriction on mobility affected the process of receiving healthcare services by stroke patients. In coping with this associated problem, the family caregivers attempted to approach the authorities, especially the police, to get permission for inter-district traveling.

*…I am not that familiar with the process because all this is considered the new norm to me… I am afraid the request for the letter will be rejected…but, I strengthen my spirit, God willing, the police signed and approved the permission…so I have no problem to travel inter-district or state to get the treatment…*

[R12, 57, wife]

### 3.6. Theme 2: Emotion-Focused Engagement

#### 3.6.1. Subtheme: The Obedience to the Strict Standard Operating Procedure (SOP) of New Norms

It was understood that the SOP was implemented to break the chain of transmission, especially when it involved gathering the public inside the premises. For most of the respondents, implementation during the early phase of Movement Control Order (MCO) was not a common practice since pre-pandemic. Although it was an unpleasant situation, the respondents eventually acknowledged the benefits and significant implementation goals. As a result, they became familiarized and capable of complying with the set procedures and accepted them as one of the new norms of the current situation. They expressed:

*…There is no mess with the new norms especially during health screening… because we already got used to it, so it’s okay…*

[R7, 35, wife]

*…It is ok, SOP has been there since last year, not a problem for me to follow…*

[R8, 59, father]

#### 3.6.2. Subtheme: Passive Response to the Burdens and Challenges of Caregiving

Underneath the emotion-focused engagement theme, most of the respondents accepted their fate regarding the conditions they faced. This coping strategy was commonly seen among the respondents and closely related to a strong belief in religious background. Having gotten used to the burden and challenges of caregiving tasks in the pre-pandemic situation, they were enabled to accept the high burden and challenges that emerged during the COVID-19 pandemic. The difficulties they faced before will empower them to cope and bear with the current challenges. Therefore, most of the respondents expressed a passive response to the burdens experienced during this pandemic. Some respondents said:

*…I have to deal with it… assuming that this is such a test from God… he wants to see how patient we are… it’s beyond my capacity… we always praise to God to make us strong and patient…*

[R3, 44, wife]

*…I hold one thing… this is the first time God tested me upon such a heavy burden; perhaps He wants to test on me… if any challenges are faced, we have to accept willingly… we should not complain about the test that has been assigned to us, God wants to grant us a reward… God only tests us on a few things…*

[R12, 57, wife]

## 4. Discussion

### 4.1. Burdens and Experiences Faced by Stroke Caregivers

The Malay ethnic group was predominant in the current study. In corresponding to the data projected by the Department of Statistics Malaysia (DOSM), Bumiputera has dominated the Malaysian citizen’s population with 69.6% out of 29.7 million citizens [41]. The respondent’s ethnic group proportion from this study portrayed the local population of Kelantan. In parallel to the recent finding by the Department of Statistics Malaysia [42], Bumiputera provided most of Kelantan’s population in 2019 (96%). The listed diagnosis of stroke survivors demonstrated a definite diagnosis, as defined based on Malaysia’s Clinical Practice Guideline on stroke.

Throughout this study, the impact of the COVID-19 pandemic can be depicted from a timeline-based standpoint. From pre-pandemic difficulties, as represented by the worsening pre-existing issues theme, to new issues that arose during the pandemic, as represented by the emergence of new issues theme. We do not have a choice except to face the pandemic. Hence, illustrating the precipitating issues remains an issue beyond their controls to solve the issues appropriately. Indeed, the unpreparedness for the unexpected pandemic resulted in the aggravation of the existing conditions. For instance, the ‘impact on social life’ subtheme emerged from ‘worsening pre-existing issues’ theme, reflecting the significant impact on youth, as the elderly, youth, and disabled persons were the most vulnerable populations to the pandemic [43]. Young people are commonly associated with active social life, various wants and needs, the uncertainty of life direction, and the unemployed. Several studies have shown that commitment while caring for stroke patients results in a lack of leisure time with friends and an inability to attend events [40]. Similar to the theme of life alterations by Wagachchige et al. [44], the family caregivers lacked leisure time and commonly missed attending social gatherings because the stroke patients could not be left alone to manage themselves. Consequently, the family caregivers felt more homebound. This signified the worsening condition as represented by the subtheme “A prisoner in your own home” under the theme “Difficulties adapting to the caregiving role” by Woodford et al. [45]. 

A similar connotation was quoted under the subtheme “Being a prisoner in their own life” by Lu et al. [39]. The sense of life being drawn, no chance of choosing one’s own life, loss of freedom, and lack of joy results in alleviating the caregiver’s well-being and health condition. Besides, the social caregiving support is highlighted through this subtheme, and this parallels the findings from studies that have shown that caregivers were connected to a lack of social support [46]. Strengthening social support was found to reduce caregiver burden, anxiety, and depression levels in a statistically significant way [47,48]. According to a local study, employed stroke caregivers had difficulty completing their jobs well and performing caregiving chores, which hampered their work performance [40]. Thus, the formal obligation responsibilities subtheme emergence represented the overburdened employed caregivers with additional work demands during the pandemic. The discrepancy between Movement Control Orders (MCOs 1.0 and 2.0) was the root cause of the subtheme of a tight appointment schedule. During MCO 1.0, most non-urgent procedures in healthcare settings were postponed ensuring enough medical personnel and hospital beds to accommodate COVID-19 admissions [49]. In contrast, the approach of MCO 2.0 was loosened to minimize the impact of MCO 1.0. This included the permission of citizens to the healthcare centers, thus returning the frequency of appointment and compensating for the postponement during MCO 1.0.

New issues emerged as the second theme. This theme emerged as one of the major components besides the other two themes. Throughout the subthemes indicated below, we can see the direct impact of the COVID-19 pandemic on family caregivers. Most of the impacts that were illustrated by respondents in these subthemes and themes resulted from the control measures taken, which included roadblock execution, compliance with Standard Operating Procedure (SOP) implementation, and practices. The execution of MCO in a few phases, including Recovery MCO (RMCO) and Conditional MCO (CMCO), was effective in controlling the widespread transmission of COVID-19 in Malaysia. This was demonstrated by simulation research using model analysis, which revealed a 99.1% reduction in peak cases, demonstrating the efficiency of MCO strategies in controlling outbreaks [9]. To fit the purpose, the police checkpoints were strengthened to be set up in most areas to curb unnecessary travel by the public [50], thus limiting public movements and avoiding mass gatherings that might increase the widespread transmission of the pandemic. Hence, the roadblock implementation nationwide incarnated by the coding of the limitation in mobility, limitation in leisure activities, and difficulties in getting necessities underneath the subtheme of the restrictions in mobility indoors and outdoors represent the real scenarios that happened.

The subsequent effect that emerged later on from these strains, also projected by respondents, related to the caregivers, coding of burdens increment and less enjoyment with pleasurable activities, and to the stroke patient, coding of delayed in the recovery process. Besides, the healthcare sector, among the other sectors, was unable to avoid the execution of MCO. Several procedures were changed by healthcare services to prevent the gathering of crowded people in healthcare facilities, and this was represented by a subtheme that emerged from the practice of strict Standard Operating Procedure (SOP). Consequently, the impact of these measures would be seen by responses, represented by the coding of the limited participation of family caregivers in the patient’s rehabilitation process, the time-consuming of performing certain procedures on SOP, the long waiting time for treatment consultation, and the bureaucracy of executing SOP, especially during health screening registration and the procedure of calling patients that would delay the treatment time.

However, rearrangement of scheduled appointments for some respondents from weekly to biweekly appointments or none at all for others was also one of the measures taken to fit the purpose. A similar trend was observed by Spalletta et al. [51] through a substantial decrease in follow-up appointments in 2020, where approximately 77.4% of patients missed out in the Italian outpatient memory clinic due to the government’s restrictive measures. Consequently, the patients would spend more time at home, leading to the coding of the additional role of the caregivers that complemented the role of physiotherapists while at home emerged. Active participation, close monitoring, and getting involved directly without abandoning the patients during home physiotherapy were the extra roles played by the caregivers.

In addition, the existence of COVID-19 would add teaching and monitoring the practice of patient’s self-hygiene care to the role of the caregivers. Another important element that we could observe in the emerging new issues theme was how the COVID-19 pandemic affected the external determinants that subsequently resulted in burdening the family caregivers. For instance, the shifted educational systems from school concepts to online learning had put additional responsibilities upon caregivers to manage children’s homeschooling, which brought about the financial issue of tools and gadgets provision for home-based learning. Furthermore, the COVID-19 would cause changes to the work of the supportive persons on whom the caregivers depended. In addition, the external determinants affected by the COVID-19 pandemic can be seen in the subtheme of financial constraints. The income of other family members was the source of the income of the caregivers and stroke patients and that the increment cost of necessities caused by the pandemic would further put the caregivers into financial hardship eventually.

Although the global COVID-19 pandemic brought negative impacts to many sectors, some positive outcomes were derived. This was identified by the theme of fewer burdens and challenges experienced by the family caregivers. The presence of the pandemic would bring the situation of increased accessibility to hospital car parks due to the fewer visitors and patients. This improved the hospital logistic issues that were known to be a problem in the past. According to [52], lockdowns reduced transportation activities, as people were not permitted to travel. This was further strengthened by national aeronautics and space administration (NASA) and the European Space Agency (ESA), which suggested that environmental quality improved with the reduction of NO_2_ emissions from vehicles by 30%, and the mobility index by Google was reduced by 90% across some of the countries.

For the above similar reason, the subtheme of shortened waiting time emerged from a respondent because of the fewer visitors or patients attended during appointment due to the rearrangement of schedule made. In additon, the lockdown or curfews resulted in limitations in mobility and flexibility or working schedules by the employed caregivers. Thus, more time was available for caregivers to be at home for caregiving, which indirectly strengthened the family bonding. Similar to the findings of a social impact survey conducted, significant gains in quality and quantity of family time were regarded as crucial life lessons and a positive effect of the epidemic [53].

### 4.2. Coping Strategies Taken by Stroke Caregivers

As for the coping strategies, we used two common approaches, which were also taken as themes: problem-focused engagement and emotion-focused engagement. According to Lazarus and Folkman [54], they theorized that coping strategies could be in the form of problem-focused coping and emotion-focused coping, based on its functions. Problem-focused includes those strategies that include acting on the environment (e.g., seeking support from others to solve the problem), whereas emotion-focused includes those strategies used to control one’s stressful emotions (e.g., using substances and emotional ventilation).

Throughout this study, there were three subthemes derived from the problem-focused engagement theme: the important role of supportive persons, adaptation to the changes made by healthcare services, and facilitating mobility during pandemics through permission from authorities. As has been proven by several previous studies, social support plays a significant role in reducing the caregiving burden. The greater the social support involved, the better the health outcomes of the caregivers and patients [55]. In contrast, the diminished interpersonal connection was the main factor in generating stress, as stated by King et al. [56]. In parallel with these studies, the coping strategies made by the family caregivers notably to get assistance from another person to support their strains in providing caregiving to a certain extent. Furthermore, they would act as a supportive person by themselves to accompany patients participating in leisure activities although during Movement Control Order (MCO) with such limitations in mobility to sustain the motivation-driven caregiving support to the stroke patients.

On the other issue, restriction measures would affect the attendance of patients to seek treatment at healthcare facilities. This was proven by a previous study conducted among cancerous patients [57], which stated that they had a strain to reach the hospital for their treatments because of travel restrictions. In addition, fear of the COVID-19 infection while in the hospital was also a factor that eventually led to the cancelation of the appointments given. Meanwhile, another study reported that 30% of oncology or hematological patients were found to cancel appointments during the COVID-19 pandemic. Most of the reasons are mainly the fear of contracting the virus (93%), besides forgetfulness and lack of urgency [58]. However, throughout the subtheme of adaptation to the changes in healthcare services derived from this study, even though the scheduled appointments were used to postpone, the family caregivers were responsible for making a call for the appointment as a coping strategy to avoid constantly defaulting and missing the physiotherapy sessions. Similar to the initiative in continuing medications, the caregivers would also make a call for a prescription. Hence, the rehabilitation process would be ensured of not being affected even with the presence of the pandemic.

However, regarding the issue of limitation in mobility and travel restriction, the application was made by family caregivers for inter-state or inter-district travel’s permission to facilitate their mobility to seek the medical attention. This was further emphasized by the official statement of the police [59] about the requirement to fill in the MCO permit forms; however, subsequently, the order was loosened to only provide medical appointment cards to pass the roadblocks [60].

Meanwhile, as for emotion-focused engagement, two subthemes were identified: obedience to the strict Standard Operating Procedure (SOP) of new norms and passive response to the burdens and challenges of caregiving. The emotion-related coping strategies associated with both subthemes were acceptance. Although some challenges might differ from the pre-pandemic era or might even be unsettling and worsening issues, the previous experiences of the burdens and challenges of caregiving would eventually make the family caregivers reach the stage of acceptance. Following the previous study, acceptance and religion were the most emotion-focused coping strategies that were commonly used by family caregivers in the caregiving of stroke patients, followed by distraction positive, denial, or blame, and distraction negative [61]. This trend was best explained because the caregiving of stroke patients was about caregiving of chronically disabled patients in the long term; thus, acceptance was considered the last stage of human psychology, and usually, it was also associated with the religious belief in continuing the care till the end. However, the practice of SOP as the new norms undeniably was accepted as the normal daily practice even if they were forced to obey initially; they subsequently harmonized without pressure. Nevertheless, to a certain extent, the public obedience toward the strict and several kinds of Standard Operating Procedure (SOP) that were introduced might be an issue in the long term, possibly inconsistency, which is termed “pandemic fatigue” [62].

### 4.3. Limitations of This Study

Similar to many other studies, this study had several limitations. This qualitative research was conducted only at two public rehabilitation centers in Kota Bharu, Kelantan. Thus, this research only targeted the family caregivers of stroke patients under two rehabilitation centers in Kota Bharu, Kelantan, and the finding might not be generalized to other settings in Malaysia or overseas. An exception is only available to the population of caregivers with almost similar characteristics of demographic, social, culture, value, belief, and with almost similar rehabilitation centers settings and practices. Besides that, the activities of daily living (ADL) of stroke patients did not take into account during participant selection. Hence, it limits the perspective views from the caregivers at a different degree of the stroke patient’s dependency as this element is strongly related to the stress and burden of caregivers. The caregivers were also interviewed only once due to the time, logistic, and technical constraints because of the pandemic; hence, the respondents might not have given full information during the session. Moreover, the local dialect being used during the interview potentially led to the interpretation of a different perspective or probably a deviation from the true meaning of the message conveyed. A similar problem was elicited in the passage through the translation of local dialect language, transcription data, or wholly the process of data analysis.

## 5. Conclusions

Through this study, we gained a better understanding and a clearer picture of what happened during the COVID-19 pandemic. The pre-existing issues involving social life, formal duty, and appointment schedule worsened with the pandemic, whereas the emergence of new issues experienced could be summarized mostly due to the implementation of control measures, especially regarding the travel restrictive measures, the execution of SOP, the adaptation made by health sectors, and the chain effects of the pandemic upon external determinants that eventually affected the caregivers. However, the COVID-19 pandemic might have positive impacts from a different angle. Like many other studies, both problem-focused and emotion-focused engagement were coping strategies commonly used by caregivers. Throughout these findings, we found that in-depth interviews aided in the clarification of fundamental issues, pinpointing the strategies that assist the sustainability and assessed the functionality of stroke healthcare services, especially during any health-related crisis. The problem raised by family caregivers in managing stroke patients could be eased by having an integrated healthcare system involving all parties during the pandemic. Sustainability of care should be put as a main priority, and it should not be compromised. The communication between family members, healthcare staff, and local authorities, such as police, should be in place and efficient to reduce bureaucracy and fasten the process. Furthermore, in the era of digitalization, we would like to urge efforts to introduce, encourage, and emphasize the consistent use of telehealth in the healthcare system to oppose the identified burdens and challenges that emerged from the issues of restrictive travel measures, strict SOP execution, the changes made by healthcare settings, and the constraints on supportive persons.

## Figures and Tables

**Table 1:** Characteristics of family caregivers and stroke survivors.

No.	Sex	Age	Ethnic	Educational Level ^a^	Employment Status ^b^	Relationship to Stroke Survivors	Diagnosis of Stroke Survivors
1	F	24	Malay	Intermediate	Unemployed	Daughter	Right MCA infarct with Left hemiparesis
2	M	43	Malay	High	Employed	Husband	Left basal ganglia bleed with Right hemiparesis
3	F	44	Malay	High	Employed	Wife	Left temporoparietal lobar infarct with Right hemiparesis
4	F	37	Malay	High	Unemployed	Wife	Right thalamic bleed
5	F	29	Malay	High	Self-employed	Daughter	Right subcortical infarct with Left Hemiparesis
6	M	25	Malay	Intermediate	Employed	Son	Recurrent stroke with Right hemiparesis
7	F	35	Malay	High	Unemployed	Wife	Left TACI with Right hemiparesis and Global aphasia
8	M	59	Chinese	Intermediate	Employed	Father	Cerebral venous thrombosis
9	F	18	Malay	Intermediate	Unemployed	Daughter	Right MCA infarct with Left hemiparesis & facial asymmetry
10	F	54	Malay	Intermediate	Unemployed	Wife	Left basal ganglia bleed with Right hemiparesis
11	M	66	Malay	High	Retired	Husband	Left MCA infarct with cerebral amyloidosis
12	F	57	Malay	Intermediate	Unemployed	Wife	Left basal ganglia bleed with Right hemiparesis
13	M	32	Indian	High	Unemployed	Son	Left basal ganglia hemorrhage with facial asymmetry

^a^ Low: elementary school or low vocational education. Intermediate: secondary school or intermediate vocational education. High: higher vocational education or university education [39]. ^b^ The employment status was categorized based on Rahman et al. [40]. F = female, M = male, MCA = middle cerebral artery, TACI = total anterior circulation infarct.

**Table 2:** Subthemes and themes on burdens and experiences in managing stroke patients during the pandemic COVID-19.

Themes	Subthemes
Worsening pre-existing issues	Impact on social life
	Formal obligation responsibilities
	Tight appointment schedule
Emerging of new issues	Restrictions in mobility indoors and outdoors
	Changes in healthcare services
	Limitation due to strict Standard Operating Procedure (SOP)
	Additional patient-care responsibilities
	Constraints of supportive persons
	Financial constraints
Fewer burdens and challenges	Improvement in hospital logistics issues
	Strengthening the family bonding

**Table 3:** Subthemes and themes on coping strategies in managing stroke patients during the pandemic of COVID-19.

Themes	Subthemes
Problem-focused engagement	Adapting available support
	Adaptation to the changes in healthcare services
	Permission to mobilize during the pandemic
Emotion-focused engagement	The obedience to the strict Standard Operating Procedure (SOP) of new norms
	Passive response to burdens and challenges of caregiving

## Data Availability

The data presented in this study are available on request from the corresponding author. The data are not publicly available due to privacy.

## Appendix A

### The Interview Guided Main and Probing Questions

**Table A1:** Examples of Main and Probing Questions.

No.	Directions	Questions	
1.	General	Main	Can you elaborate on what is your typical role in managing stroke patients?
		Probing	What are your daily routines during the caregiving of stroke patients?
2.	Burdens & Experiences	Main	Could you please explain more regarding your previous experiences during the caregiving of stroke patient? And how about during the pandemic of COVID-19?
		Probing	What are the difficulties that you need to face during the caregiving of a stroke patient?
			What are the differences in difficulties during the caregiving of a stroke patient before and after the pandemic?
		Probing	From your point of view, what do you think about the stroke healthcare system during the COVID-19 pandemic? And how about your engagement, especially on medical attention and continuation of therapies?
			How about your financial constraints on caregiving of a stroke patient especially during the pandemic period?
			How do you deal with the issues of transportation and mobilization of a stroke patient during the MCO period?
			How about your health status or even your self-health care due to most of your time spent on the welfare and caregiving of stroke patient during the pandemic?
3.	Coping strategies	Main	How do you cope with all the difficulties that you faced during the pandemic?
		Probing	How do you solve the problem that regards financial burden as you mentioned earlier?
			What about the issues on transportation and mobilization of a stroke patient? How do you cope with it?
			How do you cope with your health status and self-health care as you have mentioned earlier?

## Appendix B

### List of Initial Codes

**Table A2:** Subthemes and themes on burdens and experiences in managing stroke patients during the pandemic COVID-19.

Themes	Subthemes	Initial Codes
Worsening pre-existing issues	Impact on social life	Lacking time with friends
		Constraints to get help from others
	Formal obligation responsibilities	Additional formal duty demands of frontliner cause tiredness with the caregiving task
		The unpredictable working schedule cause unavailability as a sender
	Tight appointment schedule	The more frequent-made appointment complicate caregiver
Emerging of new issues	Restrictions in mobility indoors and outdoors	Less impacted even with mobility limitation
		Restriction to spend the leisure activities
		Less enjoyment with pleasurable activities
		High burden with the restriction-induced delayed recovery process
	Changes in healthcare services	Confusion-made by frequent postponement appointment
		Repetitive rearranged to irregular appointment frequency
		Limited participation in patient’s rehabilitative therapy
	Limitation due to strict Standard Operating Procedure (SOP)	Delayed medical attention with timely-consumed procedures
		Unreasonable long-waiting time for medical attention
		Repetitive explainable against SOP-restricted mobility
		Bureaucratic authorities to grant inter-state travel permission
	Additional patient-care responsibilities	Self-registered patient’s home physiotherapy
		Directly participate in patient’s home exercise
		Self-hygiene as patient’s focus-centered care
	Constraints of supportive persons	Driving incompetence caregiver relied upon the supportive person
		Homeschooling children loaded more burden
	Financial constraints	Family member’s financial sources affected
		The increased cost of necessities
		Additional demand expenses on children’s homeschooling
Fewer burdens and challenges	Improvement in hospital logistics issues	Availability and accessibility of hospital car parks with fewer visitors
	Strengthening the family bonding	More quality family times associated with shared responsibilities

**Table A3:** Subthemes and themes on coping strategies in managing stroke patients during the pandemic of COVID-19.

Themes	Subthemes	Initial Codes
Problem-focused engagement	Adapting available support	Other’s help as a solution to the supportive person’s limitation
		Accompany patient’s leisure activities
	Adaptation to the changes in healthcare services	Phone call upon to set the physiotherapy appointment
		Self-initiative phone call for the continuation of medicine
	Permission to mobilize during the pandemic	Attempting for inter-district traveling’s permission from authorities
Emotion-focused engagement	The obedience to the strict SOP of new norms	Used to familiarized with SOP during health screening
		Harmonization with new norms to have compliancy
	Passive response to burdens and challenges of caregiving	Strong belief in a religious dimension
		Acceptance as a destiny of life
